# Time to discontinuation in routine clinical practice of the initially prescribed antipsychotic treatment in patients with first-episode psychosis

**DOI:** 10.1192/j.eurpsy.2025.1869

**Published:** 2025-08-26

**Authors:** P. Salas Aranda, C. Garcia Cerdan, C. Martin Gomez, C. Lorenzo Romo, M. C. Turrion Gomez, M. Isidoro García, J. Pérez

**Affiliations:** 1 Psychiatry, Salamanca University Healthcare Complex; 2 Unit of Psychiatry and Neuroscience, Institute of Biomedical Research of Salamanca (IBSAL); 3 Medicine, University of Salamanca; 4 Biochemistry and Clinical Analysis, Salamanca University Healthcare Complex; 5 Pharmacogenetics and Precision Medicine Unit, Institute of Biomedical Research of Salamanca (IBSAL), Salamanca, Spain

## Abstract

**Introduction:**

The CLUMP (CLinical Utility of early intervention including the 5-Step Precision Medicine (5SPM) Method) project is a translational research initiative that aims to improve adherence to antipsychotic (AP) medications and therapeutic outcomes in patients with first-episode psychosis (FEP). CLUMP seeks to apply an early intervention model of Personalised Precision Psychiatry, based on pharmacogenetics, to this clinical group. In this specific analysis, we examine time to discontinuation of the first prescribed oral AP treatments before the implementation of the CLUMP project in Salamanca, Spain, in order to determine the impact the new Personalised Precision Psychiatry model might have on it. Indeed, given the high AP treatment discontinuation rates already identified in pragmatic, randomised controlled trials including FEP patients, these data would offer additional information about such rates in real-world clinical scenarios.

**Objectives:**

1. To assess time to discontinuation for the first prescribed oral AP treatments in FEP.
2. To identify specific AP with higher retention rates, which might reflect better tolerability and/or effectiveness.

**Methods:**

This study includes a consecutive, retrospective cohort of 42 patients with FEP treated immediately before the CLUMP project implementation, who were followed for at least one year. Kaplan-Meier survival analysis was used to assess AP time to discontinuation during the first year post-treatment initiation.

**Results:**

Table 1 (Image 1) summarises median times to discontinuation and confidence intervals (CIs) for each AP. Survival curves (Image 2 and 3) depict treatment retention trends. The overall median time to discontinuation was 36 days (95% CI: 25-153 days), suggesting an overall high early AP treatment discontinuation. Results also showed very high variability across AP, with Paliperidone (264 days) and Risperidone (72 days) having longer retention times. However, most of the sample was initiated on Risperidone (71.2%), which affects the generalisability of these results.

**Image:**

**Figure:**
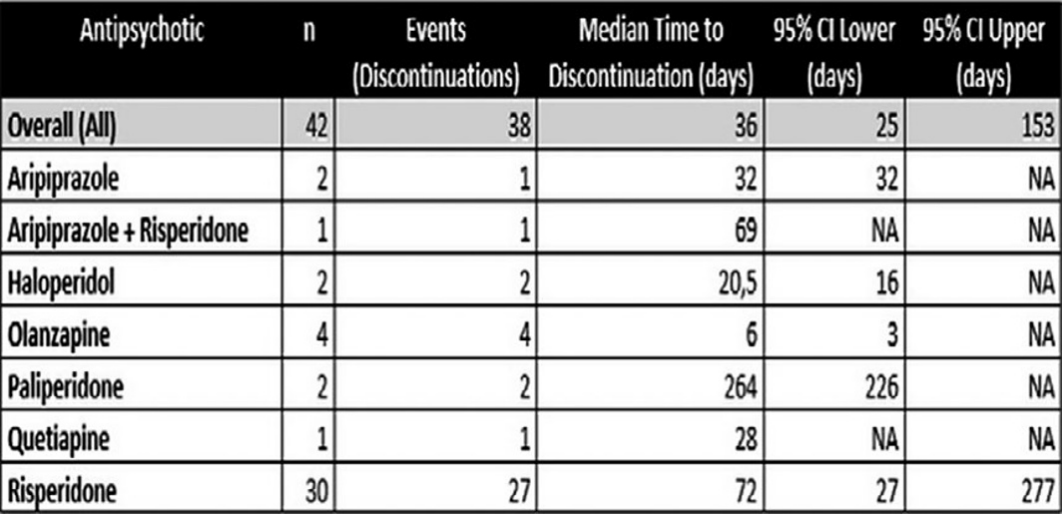

**Image 2:**

**Figure:**
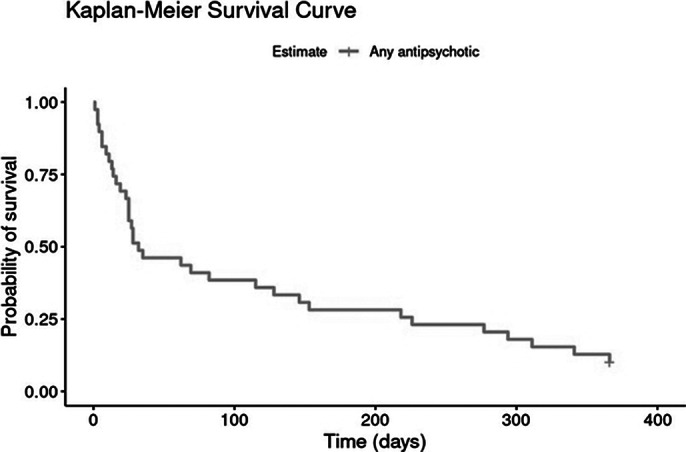

**Image 3:**

**Figure:**
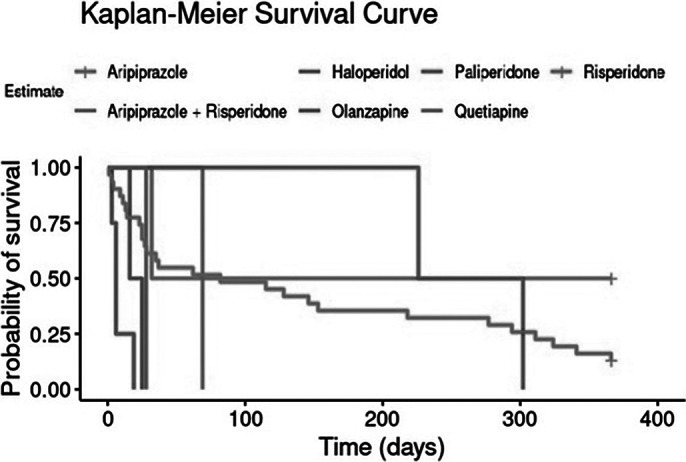

**Conclusions:**

Concurring with previous randomised controlled trials, we identified a high rate of early AP treatment discontinuation in FEP treated in routine clinical practice. For most patients, the first prescribed AP was discontinued within the first month post-treatment initiation. These results emphasise the need for a more personalised AP treatment choice for patients with FEP.

**Disclosure of Interest:**

None Declared

